# Supplementary material for: The latitudinal speciation gradient in freshwater fishes: Higher speciation across assemblages at higher latitudes in the northern hemisphere
Source: PLoS One. 2026 Jan 23;21(1):e0338966. doi: 10.1371/journal.pone.0338966 (PMC12829809; doi:10.1371/journal.pone.0338966)
Supplement: S1 Appendix — (DOCX) [file pone.0338966.s001.docx]

**S1 Appendix – Additional description of methods, metrics and datasets**

**Table S1. 1:** Means calculated in each grid-cell; each cell represents an assemblage of co-occurring species. All Σ denotes the sum over all N species (i = 1 to i = N) wi is the weight assigned to the ith species. wi= (1/number of pixels occupied by each specie).

| ^Mean^ | ^Formula^ |
| --- | --- |
| Arithmetic | $\overline{X}=\frac{\sum\lambda i}{n}$ |
| Weighted geometric mean | $\overline{X}=exp\left( \frac{\sum wiln\lambda i}{\sum\mathrm{wi}} \right)$ |

| A) | B) |
| --- | --- |

| 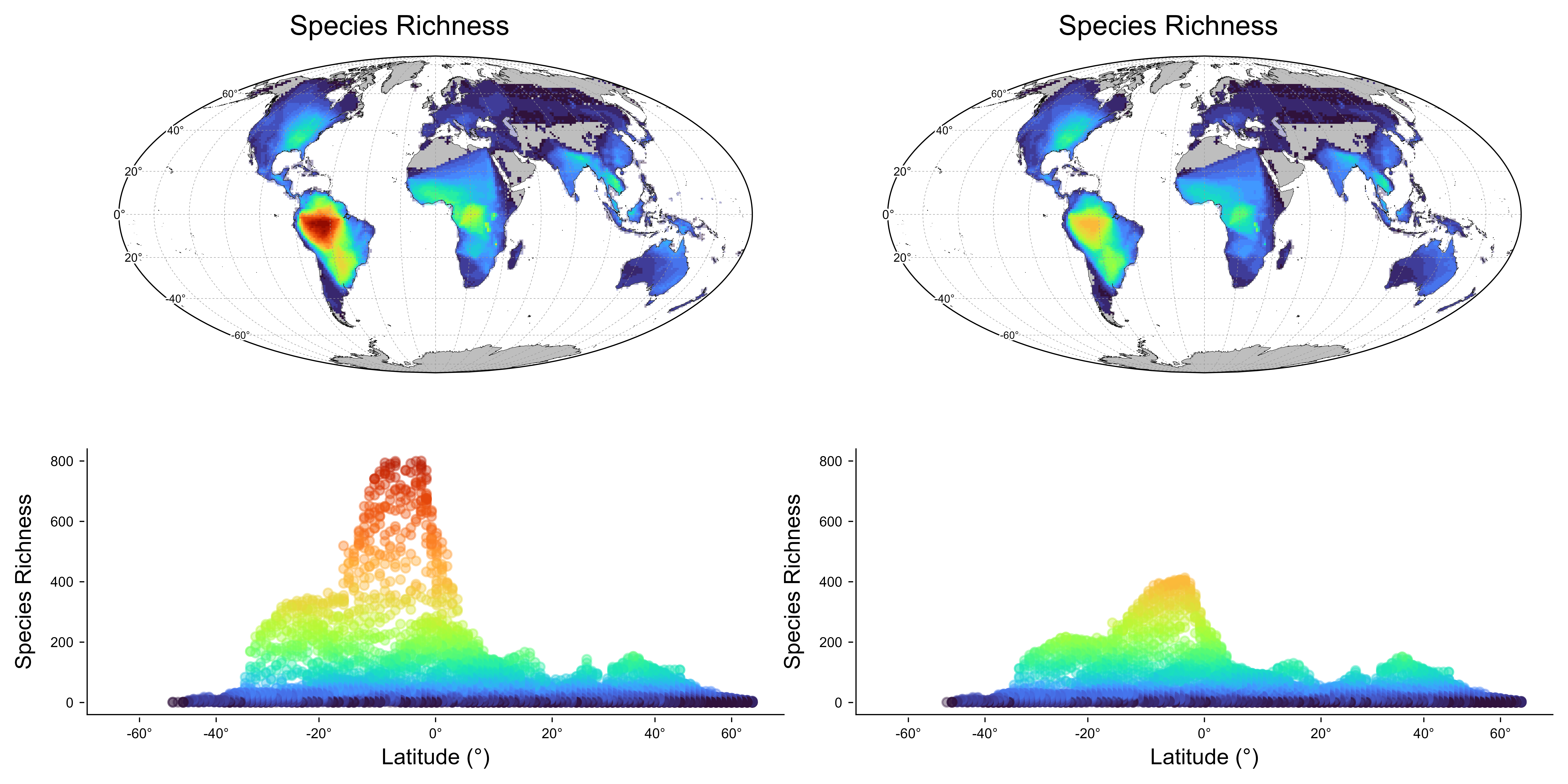 |
| --- |

**Figure S1. 1:** Freshwater fish richness pattern, A) Using the complete set of maps (García-Andrade et al., 2023) B) Using a subset with 5,242 species of freshwater fish. Maps projection is in Mollweide equal area. The maps were created by the authors in R using the open-source "rnaturalearth" package [52] with public domain data from Natural Earth (http://www.naturalearthdata.com/). The figure is published under the CC BY 4.0 license.

| 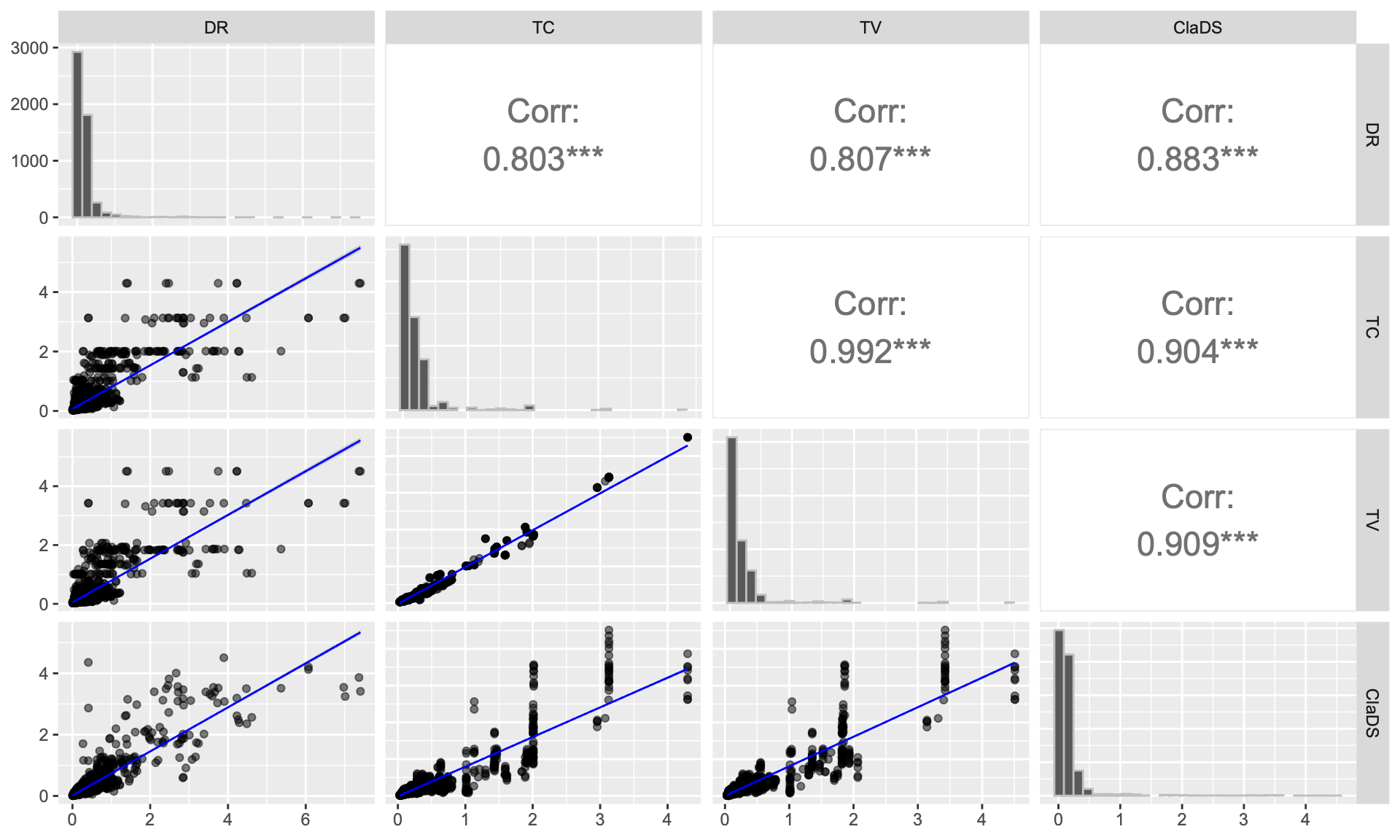 |
| --- |

**Figure S1. 2:** Species-level correlation between all speciation metrics evaluated in this study λDR, λTC, λTV, λClaDS

| A) |  |
| --- | --- |
| B) |  |
| C) |  |
| D) |  |
| E) |  |

**Figure S1. 3:** Relationship between species richness and speciation rates across grid cells. A) Complete dataset (12,577 species): species richness vs. λDR speciation rate. B,C,D) Molecular dataset (5,242 species): species richness vs. λDR, λTC, λTV, and λClaDS metrics.
