# Supplementary material for: The latitudinal speciation gradient in freshwater fishes: Higher speciation across assemblages at higher latitudes in the northern hemisphere
Source: PLoS One. 2026 Jan 23;21(1):e0338966. doi: 10.1371/journal.pone.0338966 (PMC12829809; doi:10.1371/journal.pone.0338966)
Supplement: S2 Appendix — Tables and figures corresponding to the assemblage-level results. (DOCX) [file pone.0338966.s002.docx]

**S2 Appendix. Results from the assemblage-level analysis**

**Table S2.1:** Results for the OLS and OLS-segmented model.

|  | OLS | | | | | OLS-segmented | | | |
| --- | --- | --- | --- | --- | --- | --- | --- | --- | --- |
| Dataset | Metric | Slope  (°) | R^2^ | p-value | AIC | BK | R2 | p-Davies | AIC |
| Complete | λDR | 0.0004 | 0.0071 | p<0.001 | -20392 | 47.83 | 0.0356 | p<0.001 | -20656 |
|  | λDRw | 0.0003 | 0.0029 | p<0.001 | -14957 | 47 | 0.0231 | p<0.001 | -15141 |
| Molecular | λDR | 0.0003 | 0.004 | p<0.001 | -18527 | 35.83 | 0.0152 | p<0.001 | -18622 |
|  | λTC | 0.0041 | 0.1236 | p<0.001 | -4250 | 45.27 | 0.2776 | p<0.001 | -5937 |
|  | λTV | 0.0043 | 0.1328 | p<0.001 | -4195 | 45.08 | 0.2948 | p<0.001 | -6000 |
|  | λClaDS | -1.46E-05 | 1.55E-05 | 0.7126 | -23088 | 26.31 | 0.0028 | p<0.001 | -23108 |
|  | λDRw | 0.0001 | 1.00E-04 | 0.3601 | -10039 | 31.8 | 0.0057 | p<0.001 | -10084 |
|  | λTCw | 0.0033 | 0.0652 | p<0.001 | -1740 | 46.37 | 0.1791 | p<0.001 | -2873 |
|  | λTVw | 0.0033 | 0.0649 | p<0.001 | -1619 | 46.18 | 0.1883 | p<0.001 | -2854 |
|  | λClaDSw | -0.0002 | 0.0012 | p<0.05 | -14616 | 24.39 | 0.0044 | p<0.001 | -14640 |

**Slope (1°):** Slope values transformed to decimal degrees. **R²:** Proportion of variance in speciation rate explained by latitude. **p-value:** p-value of each slope. **AIC:** Akaike Information Criterion**. BK:** Break point estimated in degrees. **p-Davies:** p-value of segmented model.

**Table S2.2:** Results for the OLS and SAR left side

|  | OLS left side | | | | | SAR left side | | | | | |
| --- | --- | --- | --- | --- | --- | --- | --- | --- | --- | --- | --- |
| Dataset | Metric | Slope  (°) | R2 | p-value | AIC | Slope(°) | R2 | p-value | AIC | Scheme | Imoran |
| Complete | λDR | -0.0002 | 0.0022 | p<0.001 | -18480 | 0.0003 | 0.5622 | 0.051 | -24499 | Wd1 | 0.681 |
|  | λDRw | -0.0004 | 0.0043 | p<0.001 | -15574 | -0.0002 | 0.3954 | 0.34 | -19216 | Wd1 | 0.548 |
| Molecular | λDR | -0.0004 | 0.0038 | p<0.001 | -13656 | -0.0002 | 0.4008 | 0.3814 | -16542 | Wd1 | 0.539 |
|  | λTC | -3.70E-05 | 2.69E-05 | 0.6732 | -12547 | 3.44E-06 | 0.4985 | 0.9873 | -17116 | Wd1 | 0.64 |
|  | λTV | -4.51E-05 | 4.34E-05 | 0.5923 | -13107 | -4.17E-06 | 0.4563 | 0.9835 | -17135 | Wd1 | 0.603 |
|  | λClaDS | -0.0004 | 0.0029 | p<0.001 | -11302 | -0.0002 | 0.4108 | 0.4406 | -13647 | Wd1 | 0.54 |
|  | λDRw | -0.0005 | 0.003 | p<0.001 | -10331 | 0.0001 | 0.43 | 0.6619 | -14026 | Sd1 | 0.57 |
|  | λTCw | -0.0004 | 0.0012 | p<0.05 | -6097 | 0.0003 | 0.4056 | 0.3825 | -9621 | Sd1 | 0.546 |
|  | λTVw | -0.0006 | 0.0029 | p<0.001 | -6140 | 0.0001 | 0.3933 | 0.8629 | -9514 | Sd1 | 0.536 |
|  | λClaDSw | -0.001 | 0.004 | p<0.001 | -5534 | -0.0003 | 0.4417 | 0.5732 | -7887 | Sd1 | 0.574 |

**Slope (1°):** Slope values transformed to decimal degrees. **R²:** Proportion of variance in speciation rate explained by latitude. **p-value:** p-value of each slope. **AIC:** Akaike Information Criterion. **Scheme:** Spatial weight and matrix distance scheme best fitted to the SAR model, chosen by the lowest AIC. **IMoran:** Moran autocorrelation using the scheme and matrix of distance in the scheme column.

**Table S2.3:** Results for the OLS and SAR right side

|  | OLS right side | | | | | SAR right side | | | | | |
| --- | --- | --- | --- | --- | --- | --- | --- | --- | --- | --- | --- |
| Dataset | Metric | Slope (°) | R2 | p-value | AIC | Slope (°) | R2 | p-value | AIC | Scheme | Imoran |
| Complete | λDR | 0.0062 | 0.0369 | p<0.001 | -2966 | 0.0101 | 0.5433 | p<0.001 | -4366 | Wd1 | 0.657 |
|  | λDRw | 0.0064 | 0.0176 | p<0.001 | -1383 | 0.0079 | 0.2926 | p<0.001 | -2000 | Wd1 | 0.472 |
| Molecular | λDR | 0.002 | 0.0131 | p<0.001 | -5399 | 0.0035 | 0.5696 | p<0.001 | -7946 | Wd1 | 0.694 |
|  | λTC | 0.0334 | 0.1454 | p<0.001 | 1028 | 0.0314 | 0.9133 | p<0.001 | -3843 | Wd1 | 0.936 |
|  | λTV | 0.0341 | 0.1505 | p<0.001 | 1055 | 0.0319 | 0.918 | p<0.001 | -3942 | Wd1 | 0.939 |
|  | λClaDS | 0.0004 | 0.0027 | p<0.001 | -1861 | 0.0011 | 0.6138 | p<0.001 | -5931 | Wd1 | 0.732 |
|  | λDRw | 0.0037 | 0.0052 | p<0.001 | -936 | 0.0043 | 0.2189 | p<0.05 | -1452 | Sd1 | 0.393 |
|  | λTCw | 0.0341 | 0.1218 | p<0.001 | 1159 | 0.0234 | 0.8346 | p<0.001 | -2107 | Sd1 | 0.887 |
|  | λTVw | 0.0351 | 0.1269 | p<0.001 | 1177 | 0.0241 | 0.8438 | p<0.001 | -2190 | Sd1 | 0.893 |
|  | λClaDSw | 0.0004 | 0.0019 | p<0.05 | -9627 | 0.0006 | 0.2862 | p<0.05 | -1196 | Sd1 | 0.46 |

**AIC**=Akaike information criterion, **Slope (1°):** Slope values transformed to degrees, **p-value**: p-value of each slope, **R^2^:** Proportion of variance in speciation rate explained by latitude, Scheme: Scheme on the spatial weight and matrix distance best fitted to SAR model choose by lowest AIC Model. **IMoran:** Moran Auto correlation using Scheme and matrix of distance in Scheme column.

| 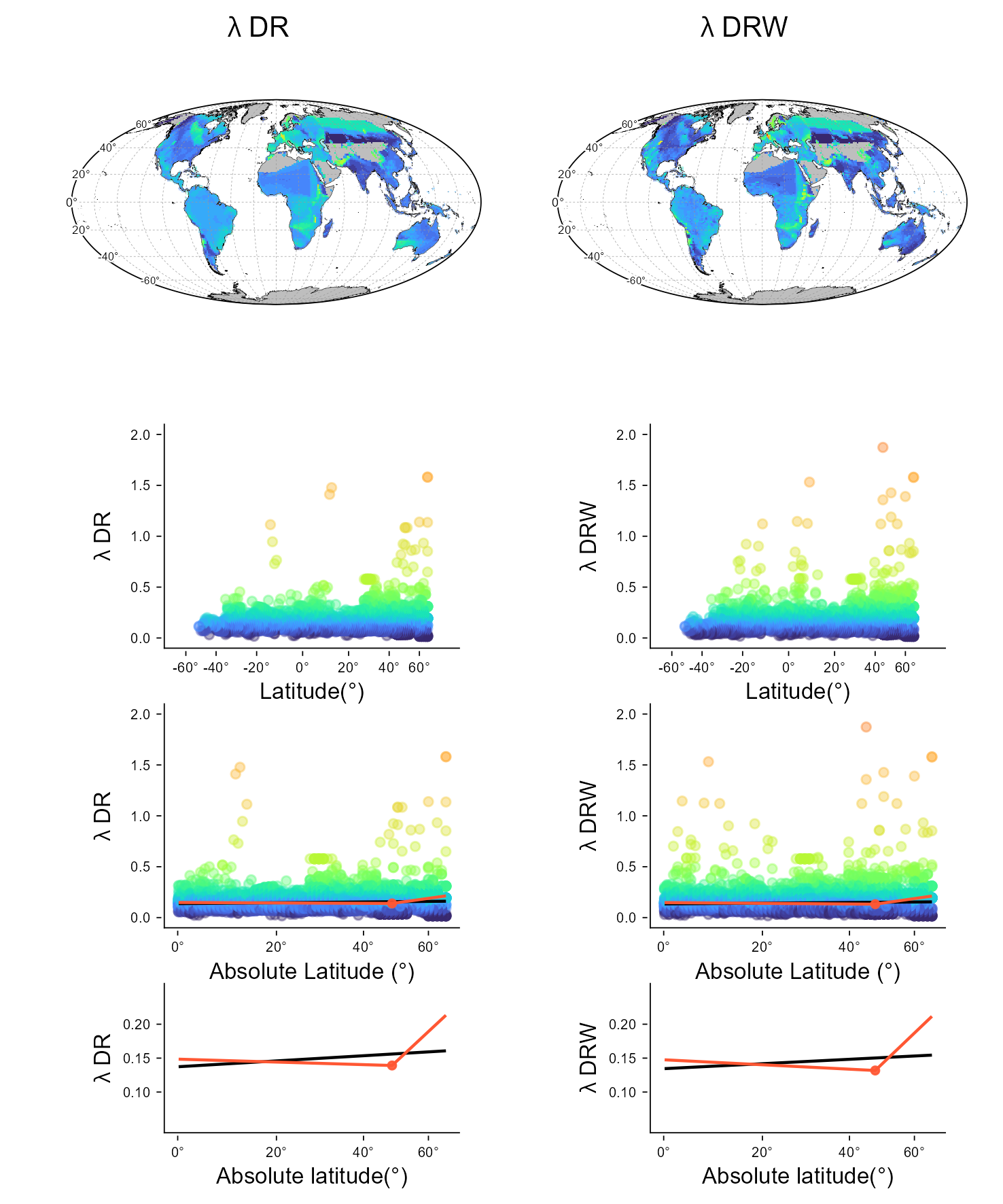 |
| --- |

**Figure S2.1**: Latitudinal Gradient in Freshwater Fish Speciation at the Assemblage Level. This figure illustrates the speciation patterns of freshwater fishes across latitudes, using a comprehensive dataset comprising 12,577 species and employing λDR and λDRw speciation metrics. Maps projection is in Mollweide equal area. The maps were created by the authors in R using the open-source "rnaturalearth" package [52] with public domain data from Natural Earth (http://www.naturalearthdata.com/). The figure is published under the CC BY 4.0 license.

The first horizontal panel displays the global spatial distribution of speciation. The second panel depicts the distribution of speciation across each pixel along the latitudinal gradient. The third panel shows the relationship between speciation and the absolute value of latitude, modelled using different adjusted approaches: the black line represents the OLS model, the orange line the OLS segmented model. The fourth panel presents the relationship zoom without data points for clarity.

| A) | 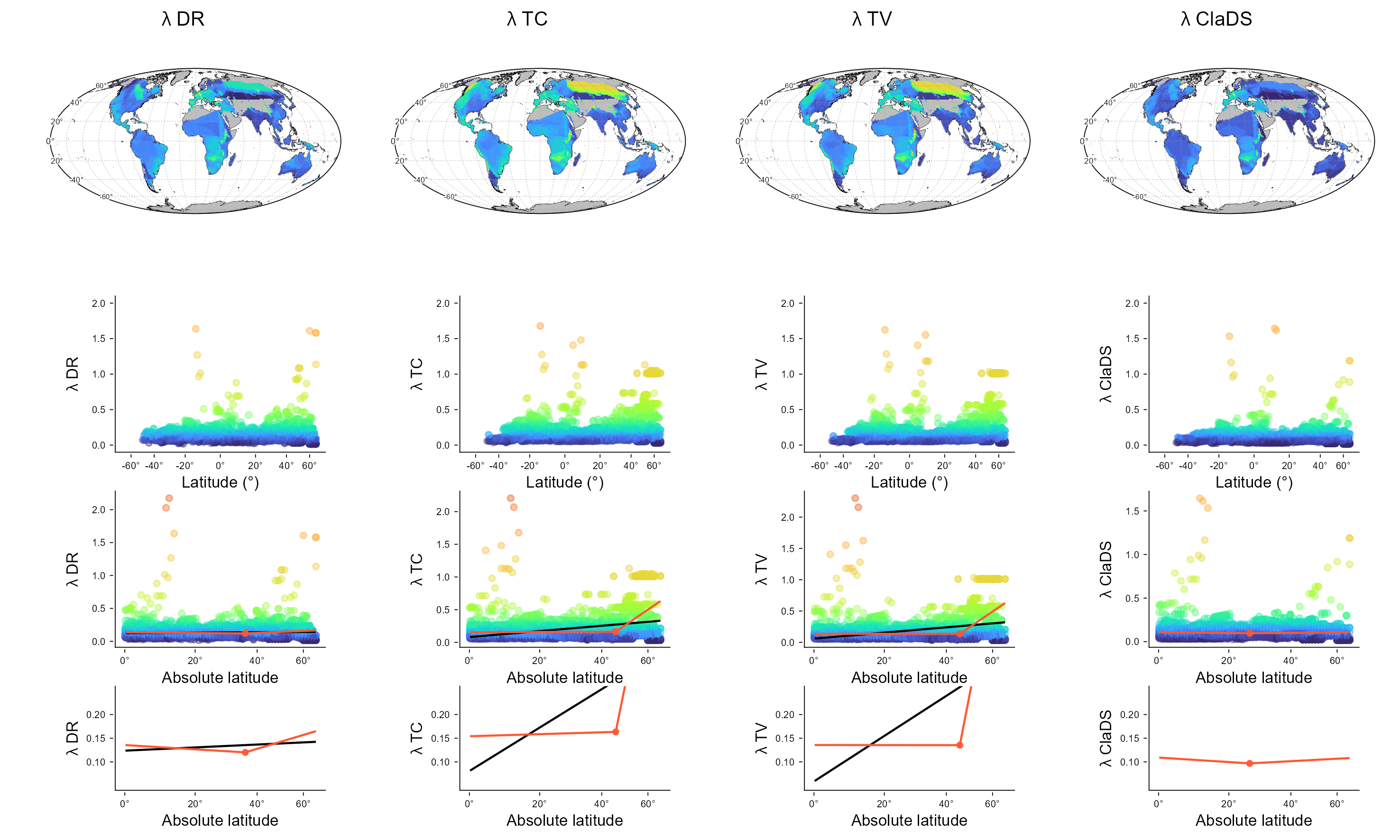 |
| --- | --- |
| B) | 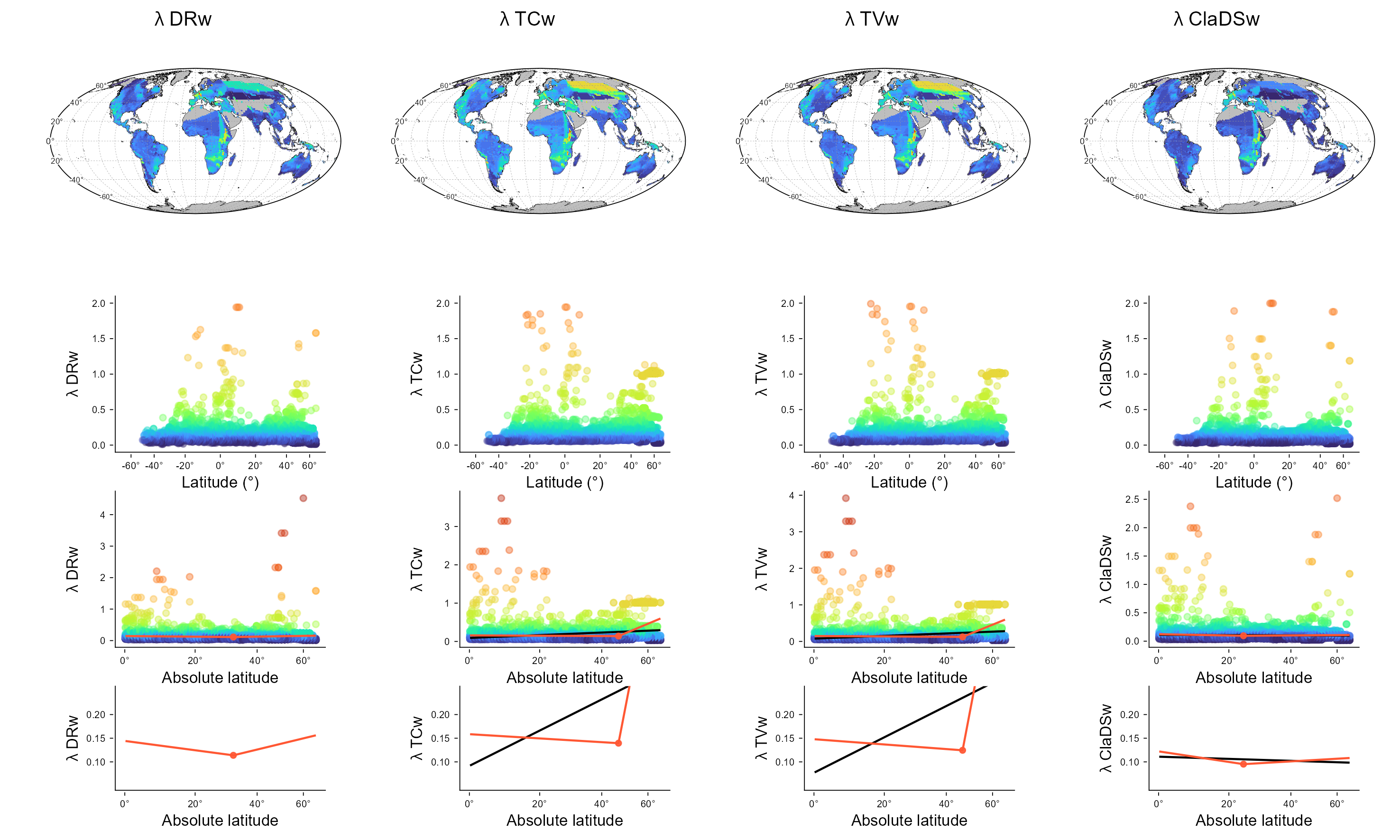 |

**Figure S2.2:** Latitudinal Gradient in Freshwater Fish Speciation at the Assemblage Level. This figure illustrates the speciation patterns of freshwater fishes across latitudes, using a molecular dataset comprising 5,242 species and employing A) λDR, λTC, λTV, λClaDS speciation metrics. B) λDRw, λTCw, λTVw, λClaDSw speciation metrics. Maps projection is in Mollweide equal area. The maps were created by the authors in R using the open-source "rnaturalearth" package [52] with public domain data from Natural Earth (http://www.naturalearthdata.com/). The figure is published under the CC BY 4.0 license.

The first horizontal panel displays the global spatial distribution of speciation. The second panel depicts the distribution of speciation across each pixel along the latitudinal gradient. The third panel shows the relationship between speciation and the absolute value of latitude, modelled using different adjusted approaches: the black line represents the OLS model, the orange line the OLS segmented model. The fourth panel presents the relationship zoom without data points for clarity.
