# Supplementary material for: The latitudinal speciation gradient in freshwater fishes: Higher speciation across assemblages at higher latitudes in the northern hemisphere
Source: PLoS One. 2026 Jan 23;21(1):e0338966. doi: 10.1371/journal.pone.0338966 (PMC12829809; doi:10.1371/journal.pone.0338966)
Supplement: S3 Appendix — Tables and figures corresponding to the species-level results. (DOCX) [file pone.0338966.s003.docx]

**S3 Appendix. Results from the species-level analysis**

| 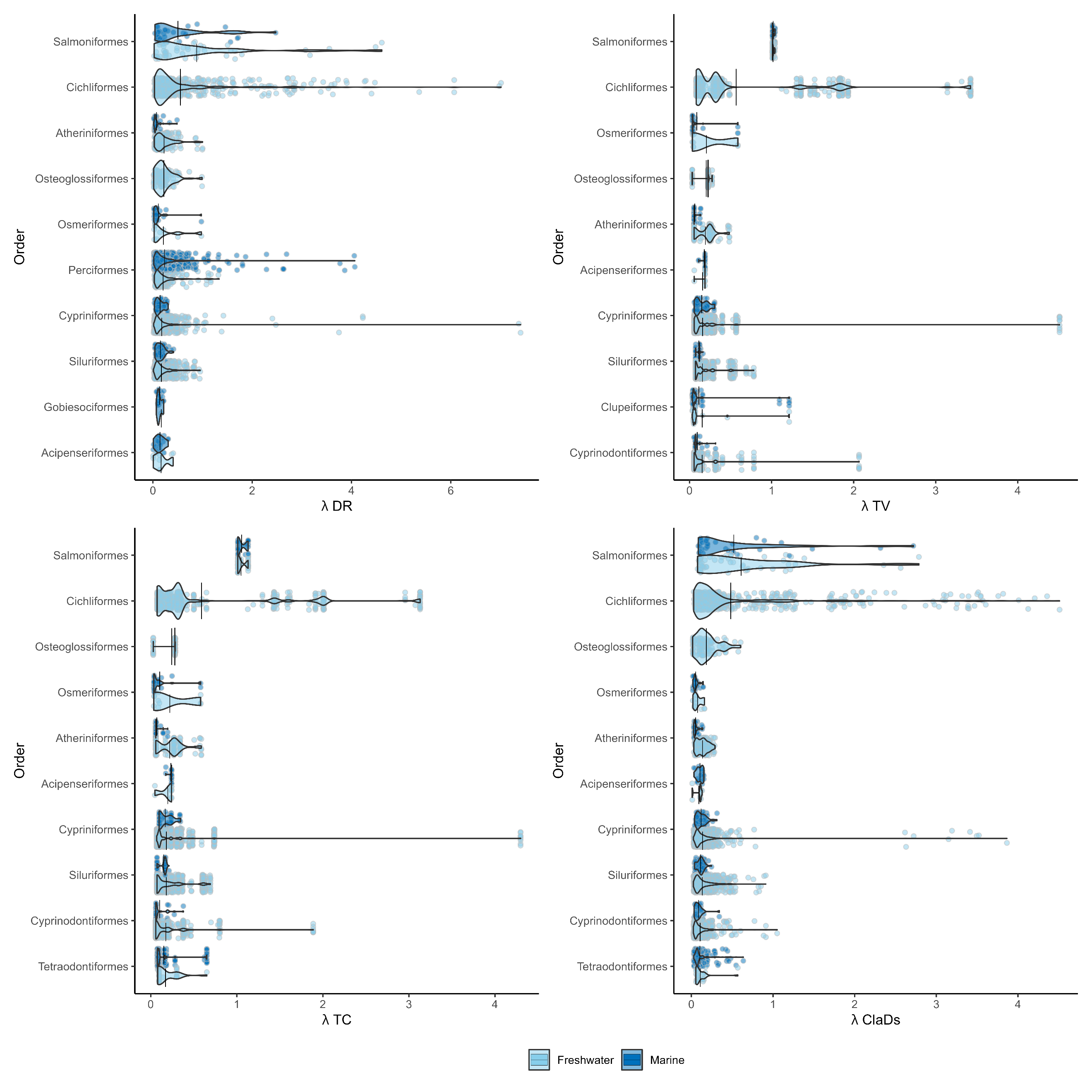 |
| --- |

**Figure S3. 1:** Speciation rates by taxonomic order, based on molecular dataset 5,242sp, comparing freshwater species with their marine counterparts.

| 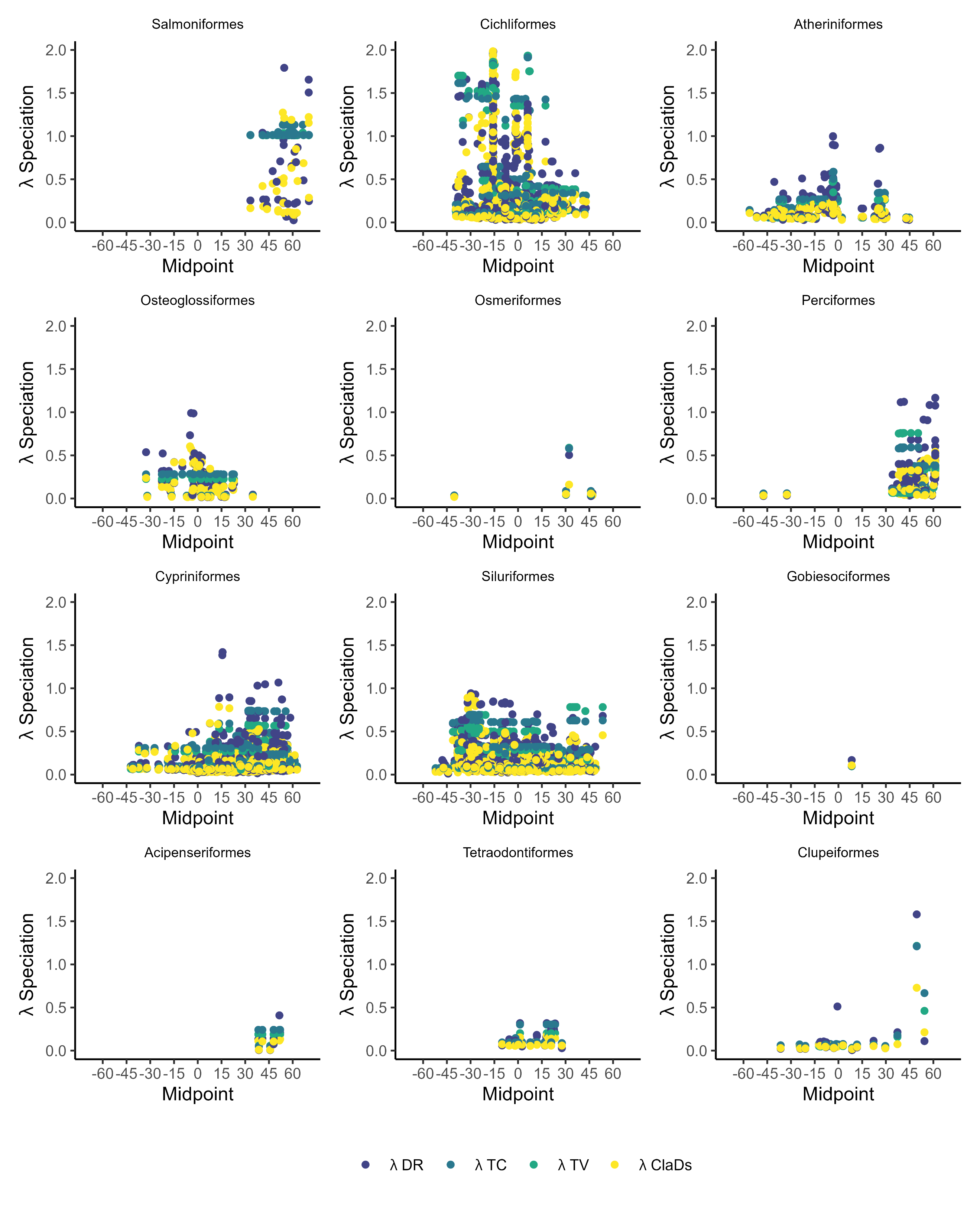 | 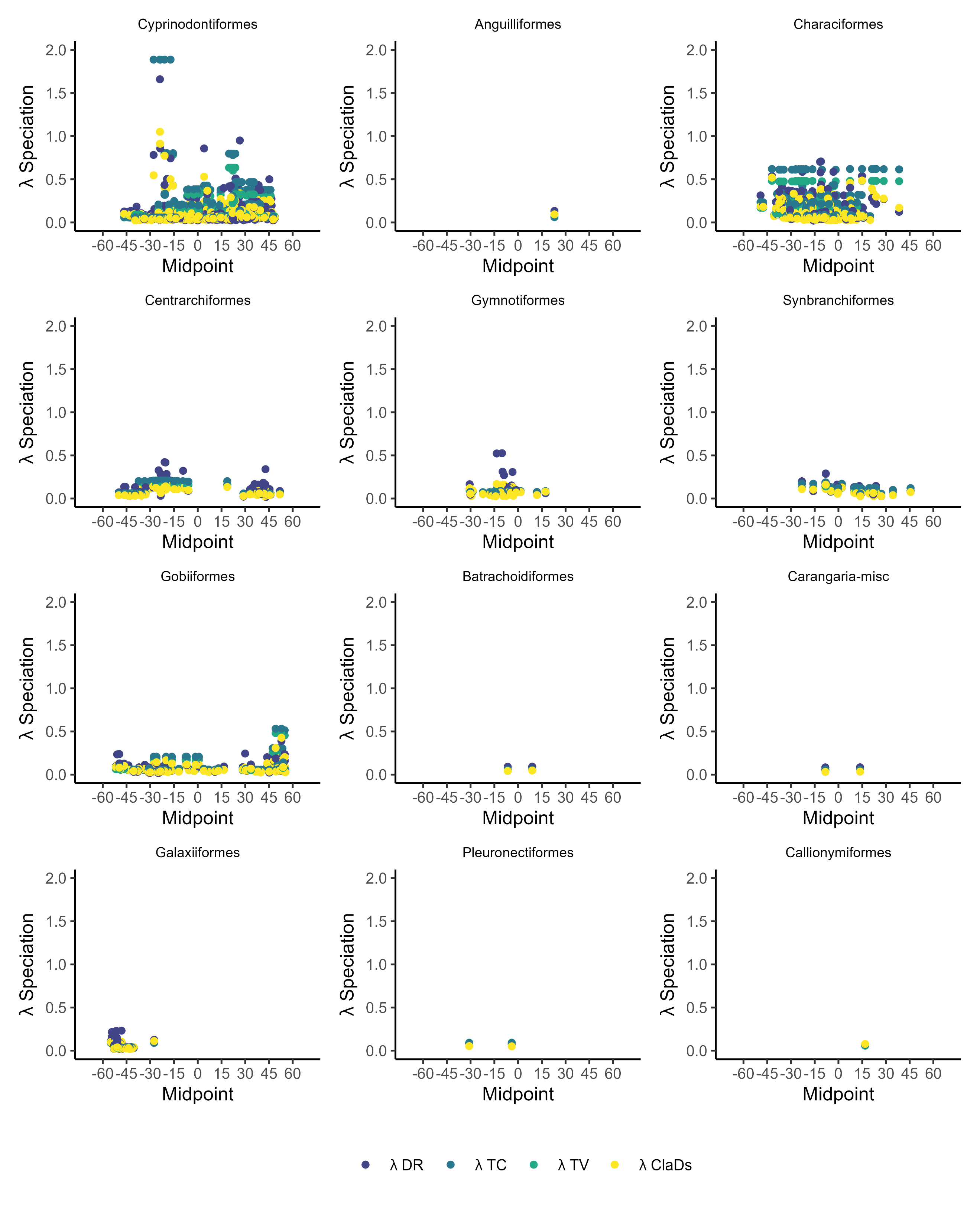 |
| --- | --- |

**Figure S3. 2:** Speciation rate λDR, λTC, λClaDS in latitudinal gradient by order, based on molecular dataset 5,242 species.


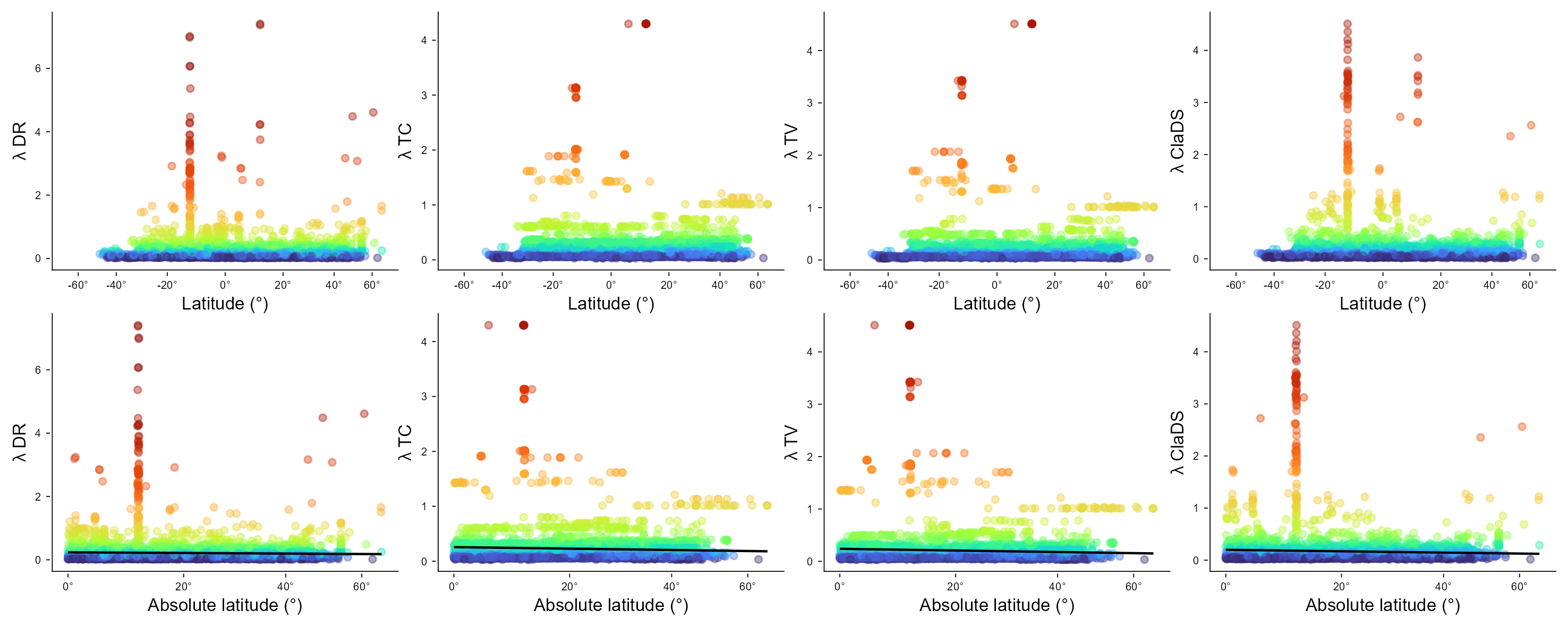


**Figure S3. 3:** Biplot latitude and speciation rate λDR, λTC, λTV, λClaDS by species in latitudinal gradient

**Table S3. 1**: Results of PGLS model using molecular dataset (5,242 sp)

|  | OLS model | | | | OLS segmented | | | | PGLS | | | | |
| --- | --- | --- | --- | --- | --- | --- | --- | --- | --- | --- | --- | --- | --- |
| Metric | Slope (1°) | R^2^ | p-value | AIC | BK | R^2^ | Davies | AIC | Slope (1°) | R^2^ | p-value | Lambda | AIC |
| λDR | -0.0008 | 9.00E-04 | p<0.05 | 6070 | 42.28 | 0.0138 | 0.76 | 6006 | 0.0015 | 0.00124 | p<0.05 | 0.75 | 3352 |
| λTC | -0.0011 | 0.002 | p<0.05 | 5093 | 42.28 | 0.0115 | 0.13 | 5047 | 0 | 3.00E-05 | 0.6722 | 1 | -4532 |
| λTV | -0.0013 | 0.0027 | p<0.001 | 5200 | 42.28 | 0.0123 | 0.25 | 5153 | 0 | 2.00E-05 | 0.7306 | 1 | -3309 |
| λClaDS | -0.0011 | 0.0026 | p<0.001 | 3896 | 42.28 | 0.0112 | 0.15 | 3855 | 0.0007 | 0.00059 | 0.0778 | 0.95 | -1779 |

**Slope (1°):** Slope values transformed to decimal degrees. **R²:** Proportion of variance in speciation rate explained by latitude. **p-value:** p-value of each slope, **Lambda:** Pagel's λ (lambda),
