# Supplementary material for: The latitudinal speciation gradient in freshwater fishes: Higher speciation across assemblages at higher latitudes in the northern hemisphere
Source: PLoS One. 2026 Jan 23;21(1):e0338966. doi: 10.1371/journal.pone.0338966 (PMC12829809; doi:10.1371/journal.pone.0338966)
Supplement: Attachment — Submitted filename: HerreraPerezEtAl-PONE-D-25-2611_R2_ResponseLetter.pdf [file pone.0338966.s006.pdf]

<u>Response Letter - PONE-D-25-26611.R1</u>

Review Comments to the Author

Reviewer #2:

The authors have done a great job in incorporating changes based on my queries and comments on the first version of the manuscript. I am happy with their responses and can recommend acceptance.

R: We appreciate the constructive and positive comments of the reviewer, which certainly helped to improve our manuscript. We are glad that our revised version met their standards and can be considered acceptable for publication.

Reviewer #3:

This is my second review of the manuscript, "The latitudinal speciation gradient in freshwater fishes: higher speciation across assemblages at higher latitudes in the northern hemisphere". The manuscript explores speciation rate at the assemblage- and species-level of freshwater fish across a latitudinal gradient. They used three different metrics to examine speciation rates, and discussed trends with OLS and an OLS-segmented models. Moreover, they examined phylogenetic structure across regions to assess potential underlying processes influencing trends. I really enjoyed reading the manuscript. It is well written, and the authors did an exceptional amount of work.

R: We are grateful to the reviewer for their encouraging comments and thoughtful criticisms. We truly believe that such careful reviews have strengthen our study.

I do have some suggestions and edits to share with the authors.
Start a new paragraph at line 160: "Phylogenetic relationships were obtained from a recent species-level phylogeny of Actinopterygian fishes…

R: Done.

I was happy to see that a flow chart was added. Unfortunately, the figure was very blurry, and I couldn't make out the words. I have a feeling it's due to the upload process.
Add a ")" at the end of "R2=0.0029, p<0.001 " in Line 327

R: Done.
Regarding the figure, yes, it must be the upload process. The figure can be accessed by clicking on the top right corner of the corresponding page where the figure was uploaded. There, there is a link to download the figure embedded in each corresponding page. Anyhow, to facilitate visualization, we are pasting a version of this flowchart figure below:

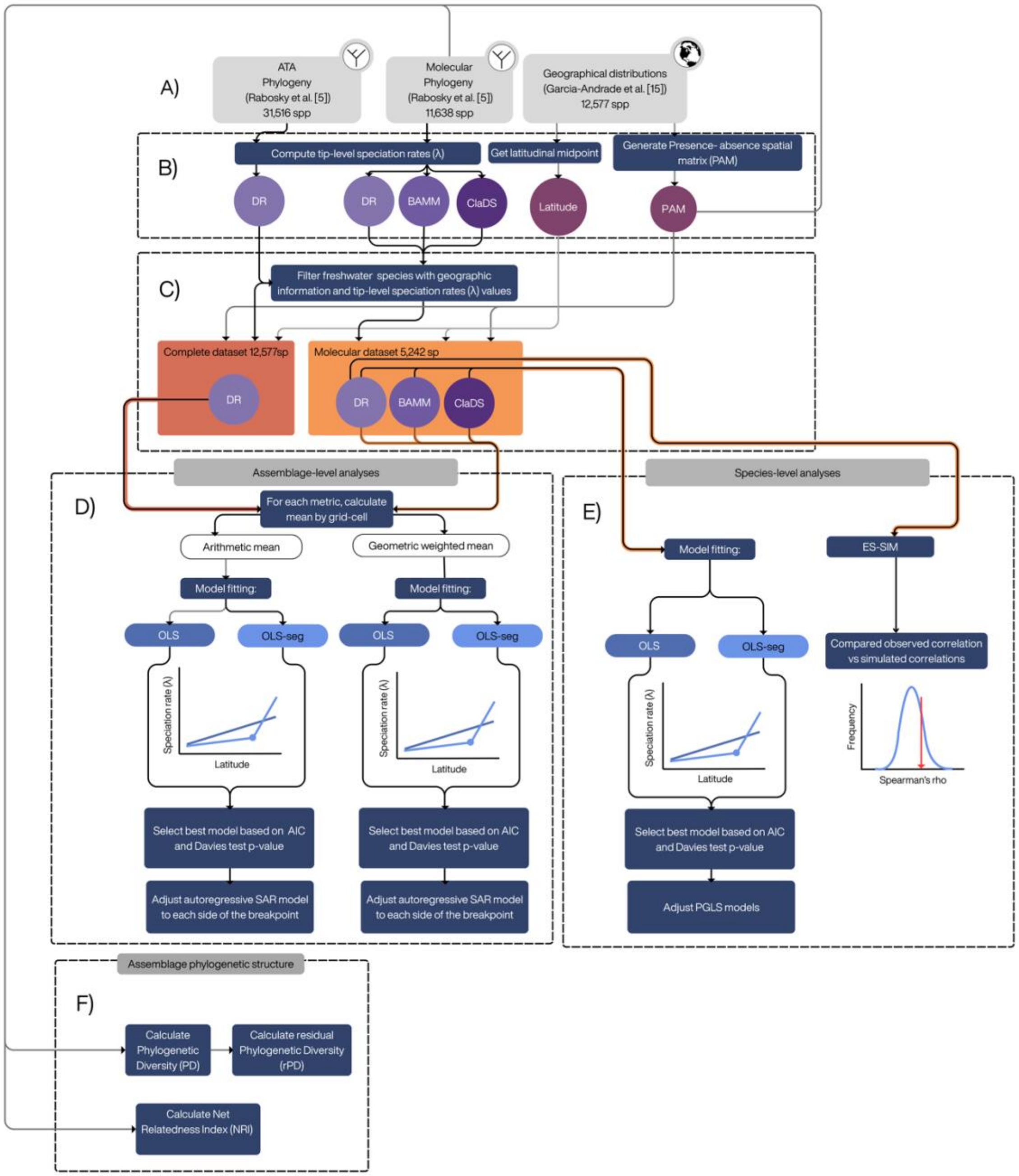
A)
ATA Phylogeny (Rabosky et al. [5]) 31,516 spp
Molecular Phylogeny (Rabosky et al. [5]) 11,638 spp
Geographical distributions (Garcia-Andrade et al. [15]) 12,577 spp
B)
Compute tip-level speciation rates (λ)
Get latitudinal midpoint
Generate Presence- absence spatial matrix (PAM)
DR
DR
BAMM
ClaDS
Latitude
PAM
C)
Filter freshwater species with geographic information and tip-level speciation rates (λ) values
Complete dataset 12,577sp
DR
Molecular dataset 5,242 sp
DR
BAMM
ClaDS
Assemblage-level analyses
D)
For each metric, calculate mean by grid-cell
Arithmetic mean
Geometric weighted mean
Model fitting:
Model fitting:
OLS
OLS-seg
OLS
OLS-seg
Speciation rate (λ)
Latitude
Speciation rate (λ)
Latitude
Select best model based on AIC and Davies test p-value
Select best model based on AIC and Davies test p-value
Adjust autoregressive SAR model to each side of the breakpoint
Adjust autoregressive SAR model to each side of the breakpoint
Species-level analyses
E)
Model fitting:
ES-SIM
OLS
OLS-seg
Compared observed correlation vs simulated correlations
Speciation rate (λ)
Latitude
Frequency
Spearman's rho
Select best model based on AIC and Davies test p-value
Adjust PGLS models
Assemblage phylogenetic structure
F)
Calculate Phylogenetic Diversity (PD)
Calculate residual Phylogenetic Diversity (rPD)
Calculate Net Relatedness Index (NRI)

While the authors do describe the differences between the speciation rate calculations in the discussion. I wanted the differences discussed sooner (methods) to gain a better understanding of discrepancies in species-level analyses.

R: Thanks for the observation. We agree with the reviewer and have now added the following sentences to the corresponding paragraph in the methods section ("Speciation rates"; lines 203-209):

"*Each of these speciation rate metrics have important differences, with BAMM identifying discrete shifts in diversification regimes (i.e., cohorts) that tend to reduce rate heterogeneity along the phylogeny compared to DR and ClaDS. Conversely, DR and ClaDS recover higher rate heterogeneity compared to BAMM, with ClaDS being able to identify many small and frequent shifts that result in more heterogeneous rates among lineages [12]. Accordingly, considering these three metrics allowed us to evaluate the robustness of our studied patterns beyond methodological differences.*"

New paragraph at line 332. "OLS-segmented models provided a better fit …"
R: Done.

I was confused why OLS results were present when OLS-segmented models were a better fit. I was making notes on the OLS results, until I came across that OLS- seg was a better fit. I suggest keeping the OLS model results in the supplemental material and focusing on the OLS-segmented results. The OLS results provide interesting information, but for the reader to stay focused, keep the results streamlined. Moreover, I would keep the AIC scores in, so the reader can see why OLS-seg was chosen.

R: We understand the reviewer's concern. Certainly, we do want the readers to stay focused, but we also want them to have the elements for considering our study within the broader literature, mainly the one related to the other main group of fishes, the marine fishes. Accordingly, we considered it important to keep the presentation of the OLS results to describe the general/global relationship between latitude and speciation rates, which is the most common in the literature being used to describe the pattern for marine fishes and other taxa (e.g. mammals, birds, amphibians). Also, we think that showing the OLS and OLS-segmented results helps to explicitly/clearly illustrate the fact that the relationship is indeed more complex than a simple linear relationship, which in turn allows a more comprehensive discussion of our findings with those of others.

Add a ")" to "(β=-0.001, R^2=0.004, $p<0.001$)" on line 349.
R: Done.

In paragraph on line 395, you discuss NRI values, but don't reference the values or a figure. Can you add a figure reference to the paragraph?
R: Done. We have included a reference to Figure 6 that shows those patterns.

Fig 6 caption doesn't say what NRI represents.

R: Done.

The authors discuss marine fishes having a more pronounced gradient in speciation in the intro and discussion, line 440, "As expected, the global assemblage-level pattern for freshwater fishes is less pronounced than that observed for marine fishes". Without concrete information on the difference between the two studies, this assessment feels qualitative and like a strawman argument. I doubt that it is an incorrect assessment by the authors, but as a reader, I need something tangible to compare the difference.

R: We appreciate the reviewer's comment and opportunity to clarify our message. We agree with the reviewer in that we need something more tangible to compare our results with those of marine fishes. Therefore, in our new revised version, we have expanded this part of the discussion to clearly relate our findings to those of marine fishes, thus providing more tangible evidence to conduct such comparison, including reference to our figures.

In particular, we added the following text to the cited paragraph (lines 445-458):

"*The latitudinal speciation gradient of freshwater fishes was less consistent than that observed for marine fishes. In marine fishes, Rabosky et al. [5] found a consistently positive global relationship between latitude and speciation, regardless of their considered metrics (BAMM or DR). In contrast, the global relationship for freshwater fishes was not consistent between these two metrics, with BAMM showing the same pattern to marine fishes whereas DR showed no significant latitudinal trend. Indeed, marine fishes exhibited a clear u-shaped pattern between latitude and speciation, with high speciation rates at both northern and southern latitudes [5]. Instead, freshwater fishes did not show such a clear pattern but a more heterogenous one with higher variation of speciation rates in tropical regions (Fig. 2). This difference between marine and freshwater fishes is mainly given by tropical freshwater assemblages exhibiting very high speciation rates, whereas tropical marine assemblages showed consistently low speciation rates [5]. Our observed speciation peaks in the tropics were located in places with high phylogenetic clustering, indicating the co-occurrence of recently derived and/or closely related lineages within the assemblages (Fig 6), which is consistent with recent findings for freshwater fishes at the basin level [4], where the highest speciation rates are located in recently colonized basins.*"

On line 444, "Our observed speciation peaks were found in places with high phylogenetic clustering", I cannot fully connect your results to this statement. Are you connecting NRI and speciation values to the Neotropic (or tropical) and Nearctic regions? Later in the paragraph, you reference regions in Africa (that you found high NRI values) and neotropics (where you found sig. NRI values), but you are connecting results from two sections, making it slightly hard to follow. The reader might need some hand-holding or references to figures.

R: Thanks for the important suggestion. In following it, we have added an explicit mention to the "tropics" and a reference to the relevant figure for this pattern (Fig 6).
