# Supplementary material for: The latitudinal speciation gradient in freshwater fishes: Higher speciation across assemblages at higher latitudes in the northern hemisphere
Source: PLoS One. 2026 Jan 23;21(1):e0338966. doi: 10.1371/journal.pone.0338966 (PMC12829809; doi:10.1371/journal.pone.0338966)
Supplement: Attachment — Submitted filename: HerreraPerezEtAl-PONE-D-25-2611_ResponseLetter.docx [file pone.0338966.s005.docx]

**Response Letter - PONE-D-25-26611**

**Reviewer #1:**

This paper examines patterns of speciation rates of freshwater fishes across latitude, globally, finding that speciation rates increased with latitude – mainly in the northern hemisphere. The aim of the study was to describe and evaluate the latitudinal pattern of speciation in freshwater fish. And, also to determine whether speciation rates were related/responsible for the global pattern of species richness – higher richness at the equator − but this was not explicitly stated.

R: We appreciate the reviewer’s feedback and suggestions to improve our manuscript. The reviewer correctly highlights our main goals. Since we were mainly interested in describing and evaluating the latitudinal gradient of speciation in freshwater fish, which has not been previously done in the literature, we focused on this explicit goal while its relationship with the global pattern of species richness was left as part of our motivation and discussion. However, we agree with the reviewer that we should also explicitly state this secondary goal. Of course, speciation rates are ultimately related to species richness but evaluating their interdependence requires a completely different approach beyond the scope of our study. Instead, we considered such relationship between species richness and speciation to base our expectations about the latitudinal speciation gradient, following the relevant literature on the general topic of speciation gradients (Schluter & Pennell 2017, Nature) and for fishes in particular (e.g., Rabosky et al. 2018, Nature). Following another reviewer’s suggestion, we now explicitly included the relationship between species richness and speciation rates as part of our goals, and added a plot (now Figure 3) to support our discussion and interpretation.

Speciation was evaluated at the assemblage- and species-level using various metrics of tip-level speciation rate and multiple models. Findings indicated the importance of glaciations/refugia, biogeography, and evolutionary factors on speciation gradients. Where speciation was high, rates were congruent with recent radiation events of specific taxonomic groups; dispersal and environmental filters related to glaciation events likely played an important role here. This is an interesting paper, which highlights the importance of fragmentation and environmental filters on diversity.

R: We thank the reviewer for the positive appraisal of our study and the constructive comments to improve it.

The material in the introduction and discussion is well written and clear with supporting citations. My main criticism – albeit a large one – is that the methods are unruly, hard to follow, and poorly justified. I think much of this can be corrected, and as clarifications are made, I believe some of the other more minor questions I had in the discussion will be addressed.

I think the paper would benefit substantially from a flow chart that demonstrates how all of data and the models came together and how choices were/are made. There are numerous streams of information including two pattern levels, two datasets, multiple metrics of the tip-level speciation rate, several types of model structure, multiple mean calculations, and transformations. This is very complicated, and I cannot make sense of why certain things were done (e.g., the use of GAMs in addition to the other model types).

Instead of running all the models on all the metrics and showing all the results, I suggest narrowing the focus. What is the main question you are asking in this paper? What model (GAM, breakpoint, …) best fits the data and answers the question? In the discussion as it is currently written, most focus is on break points. Is this the best model tool?

R: We understand and agree with the reviewer’s concern. By trying to robustly address our main goal, we inadvertently complicated things more than necessary. In our revised version, we have carefully selected and appropriately justified our methodological choices.

First, we now provide a detailed flowchart in the main text (now Figure 1) describing how the data and models were structured based on our specific choices with their corresponding goals.

Second, given that all metrics, their different versions and transformations considered in our original manuscript showed basically the same patterns, we have reduced the number of metrics of tip-level speciation rates. More specifically, we kept the raw metrics from the three different methods (λDR, λBAMM and λClaDS) to ensure robustness of our results across distinct methods and discuss such robustness in light of recent debate regarding these methods (as suggested by other reviewers). Also, these raw metrics allow easier interpretation and comparison with previous studies relevant to ours, such as the latitudinal speciation gradient of marine fishes (Rabosky et al. 2018). In addition, for the assemblage-level analyses based on mean calculations, we also included the weighted geometric mean versions of the raw metrics, mainly because these metrics versions are less sensitive to extreme values and, more importantly, control for the disproportionate contribution of widely distributed species across different assemblages.

Third, we have narrowed our focus to our main question and goal, describe the latitudinal speciation gradient of freshwater fish speciation rates and assess whether there is a consistent relationship across the gradient or a breakpoint as suggested for marine fishes. Accordingly, we kept the breakpoint models (segmented regressions) while comparing them to OLS models to confirm the existence of a breakpoint (i.e., better model fit), followed by the SAR models fit to the relationship at each side of the breakpoint to explicitly account for spatial autocorrelation in the assemblage-level analyses. We followed the same procedure for the species-level analyses, comparing OLS vs segmented models and, after confirming that OLS models consistently provided a better fit, we fitted a PGLS model to account for phylogenetic non-independence among species.

As such, we removed the GAM models that were initially considered solely to confirm that the studied relationship was not linear, but this is already accounted for with the comparison between OLS and breakpoint models.

With these adjustments, we strongly believe that our methods are now clearly justified and easier to follow.

I am also confused by the use of AIC, and not just because there were so many models. If I look at any of the given tables in the supplement, a lower AIC score is often found for a model that was not reported, and AIC is seemingly not used for model selection. For instance, the AIC for the global ordinary least squares model (line 294) is not the lowest score on the associated table (suppl Table S2-1), it is actually for log(λDR-geo) (AIC = -25043) not for λDR (AIC = -20392). Why use AIC if you are not going to consider the best fit model? This occurred across models.

R: We apologize for the confusion and thank the reviewer for the opportunity to clarify our approach. Originally, our approach was to compare models only within the same type of speciation rate metric (e.g., OLS vs Breakpoint models for λDR) as these have the same data structure, which is a requirement for valid model comparison and selection (Burnham & Anderson, 2002). In other words, it is not appropriate to compare a model for λDR with a model for λDR-geo since they are essentially different response variables (Burnham & Anderson, 2002). To avoid confusion, we now explicitly mention this methodological aspect while also simplifying things by reducing the number of considered metrics (see previous response). In short, we used AIC-based model selection to evaluate whether the breakpoint model was a better fit than the OLS model. For the assemblage-level analyses, we further compared the fit of OLS vs SAR models to the relationships at each side of the breakpoint. For the species-level analyses, we compared the fit of OLS vs PGLS models.

I also lost track of the methods for the result. In some cases, I don’t know that there was a method written, for example the correlations among the different metrics λ-DR, λ-TC and λ-ClaDS at the species-level. Also, if they are all strongly correlated, why not pick one? Why continue to show all results? On lines 527-534 the caveats of the different metrics are briefly discussed – this would be more useful in the analyses as a tool for helping select a metric in the methods. If you do keep using more than one, emphasize how it effects the interpretation.

R: The reviewer is correct in that the correlations were not described in the methods section. We now include this methodological step. In this sense, we decided to keep the speciation rate metrics derived with different methods to ensure robustness of our results across distinct methods and discuss such robustness in light of recent debate regarding these methods. We now include more explicit discussion on this issue as suggested by other reviewers.

Still, we did reduce the number of total metrics considered, keeping only the raw metrics and their weighted geometric mean version (for the assemblage-level analyses). Please, see our response above on this issue.

These issues with the methods need to be addressed to improve the clarity of the paper and really drive home the point without the distraction of three or four models that provide the same end. I offer other suggestions and editorial comments in the notes below.

R: We thank the reviewer for providing specific guidance to improve the clarity of our study.

Line 69: Define tip-level speciation rates

R: Thanks for the suggestion. We now explicitly define tip-level speciation rates. The revised text now reads (lines 66-70):

*“For example, different metrics with different assumptions have been proposed to estimate tip-level diversification rates, which can be interpreted as estimates of present-day rates of speciation or extinction of individual lineages/branches conditional on their past evolutionary history but provide more reliable estimation of speciation rates instead of net diversification [11,12].”*

Line 77: Delete the space between the citation and the comma and put a period at the end of the sentence.

R: Done.

Line 83: Clarify here, “that limit connectivity” do you mean limit the flow of genetic material or that fragmented habitats have limited connectivity?

R: Thanks for pointing this out. Yes, we referred to limited spatial connectivity in fragmented habitats. We added the term ‘spatial’ to this part of the text, which also helped connect with the next paragraph in terms of how the dendritic network limits movements and thus affects processes such as dispersal and speciation.

Line 93: freshwater habitats are more susceptible than what? Marine habitat?

R: Yes. We now clarify that comparison with the marine habitat.

Line 95: Period needed after the citation.

R: Done.

Line 116-121: I am confused, here, the sentences seem almost contradictory and with details that should in the methods. Maybe edit, “This study focused on species richness explanations at the assemblage and species level, capturing detailed variation among species and across space, thus helping untangle clad-specific or region-specific patterns.”

R: We agree with the reviewer that our phrasing was confusing. In the first sentence of this part, we were actually referring to the study by Miller & Román-Palacios (2021) [reference #4] cited in the previous sentence. We now explicitly cite this study in this part to avoid the confusion. The second part of the text referred to our proposal of combining species- and assemblage-level analyses. We kept this part to explicitly state the difference between previous studies (Miller & Román-Palacios 2021) and our proposal. The revised text now reads (lines 112-116):

“*Similar findings were obtained by Miller and Román-Palacios [4] for freshwater fishes when evaluating their latitudinal species richness pattern, with higher species richness in tropical basins associated to low speciation rates and low species richness in basins with high speciation rates. However, the study by Miller and Román-Palacios [4] focused on species richness explanations…*”

The advantage of using both the assemblage- and species-level speciation is stated as capturing more variation, but the reasoning is unclear. Please clearly indicate: What is the advantage of an assemblage-level speciation investigation? And what is the advantage of a species-level speciation investigation?

R: Thanks for the suggestion. We now provide a clearer reasoning on using these two approaches, clearly stating their advantages. The revised text now reads (lines 119-124):

*“Species-level analysis allows identifying lineage-specific patterns by considering individual species as observational units and describing trait (here, speciation rates) variation across the clade. Complementarily, assemblage-level analysis allows describing the entire geographic structure of mean trait variation by considering assemblages of species across space [29]. Thus, combining both types of approaches helps untangling clade-specific from regional-specific patterns [29].”*

Line 122: In this paragraph, add one or two sentences clearly emphasizing the importance of this study. Why is it important to gain insight to evolutionary process of speciation, extinction, and dispersal?

R: Done. We appreciate this important suggestion. We added statements highlighting the importance of studying speciation rates and their geographic variation to understand biodiversity gradients, especially species richness differences among regions (Schluter & Pennell 2017, Nature), as well as describing spatial patterns of phylogenetic structure to infer the roles of the evolutionary processes that ultimately cause such biodiversity gradients. The revised text now reads (lines 128-131 and 137-142):

“*To provide different perspectives on the same pattern and a more robust evaluation, we employed both assemblage-level and species-level analysis [29,30] of the geographical variation in speciation rates, which can help infer its role in explaining the observed LDG of freshwater fish species richness [5,8].*”

“*These patterns can provide insights into the maintenance of biodiversity gradients by describing the roles of the evolutionary process of speciation, extinction and dispersal, which are the ultimate causes underlying such gradients [8, 31]. Recent studies have evaluated these phylogenetic structure patterns in freshwater fish but focusing on specific regions independently (e.g., North America [32]; South America [33]), thus a global description is still lacking.*”

Line 150: Anadromous/catadromous instead of migratory? Since there are species that migrate fully in freshwater.

R: Done.

Line 363: I do not understand the motivation for excluding the Cichliformes. Is there a biological or ecological reason to do so? Do they represent some type of oddity? It makes no more sense than excluding Salmoniformes. Cichliformes speciation is a good point for discussion and should continue be presented there.

R: Our motivation was not to neglect the importance of Cichliformes but instead consider their disproportionate effect on the evaluated pattern, which was evident when we excluded them from the whole-class analysis. In fact, we considered and discussed both patterns with and without Cichliformes in our previous version. Nevertheless, we agree with the reviewer (and the other reviewers) in that there is no a priori reason to do so and that our results should be discussed in light of their presence not its (artificial) absence from the whole-class global pattern. Accordingly, in our revised version we only kept and discussed the analysis/results considering Cichliformes and thus freshwater fishes from the whole class Actinopterygii.

Line 447: Myr ago. Also, in some places it is “Myr” and in other “myr”

Done.

Line 449: delete “higher”

Done.

Line 450: “Similarly” instead of “In the same vein”

Done.

Line 451: “genera” instead of “groups”

Done.

Line 453-454: This sentence is awkward; it reads as though the analysis in this paper examined speciation of marine fish. I suggest: “… environments only occur outside the tropics, between 27° and 40° latitude [5]. Among freshwater fishes the latitudinal position of…”

R: Thanks for the suggestion. We followed the reviewer’s suggestion and modified our sentence accordingly.

Line 460: reference?

Added.

Line 498: “find evidence of” instead of “not recover”

Corrected.

Line 520: define the Wallacean and Darwinian shortfalls

Done.

Figures: All figures are blurry, and quality should be enhanced.

R: We apologize for this issue, but we think it is related to the compilation of the PDF document by the journal’s system that reduces their quality. We also submitted high resolution figures as separate files that should be available in the journal’s system. You can download each figure by clicking on the top right corner of the corresponding PDF page.

Fig2: I cannot differentiate the lines, perhaps show below as in figure S2-5

Corrected.

Fig 4: write out “Next Relatedness Index” in the figure caption

Done.

Given the interest in speciation rates as related to species richness. I’m curious what this plot would look like, with species richness on the x-axis (instead of latitude) and speciation on the y-axis. Perhaps that would show an interesting pattern, maybe you’ve already considered this, and it doesn’t provide anything useful. Just a thought.

R: This is a great suggestion, thanks. We have now prepared this plot and provided it in the supplementary material as figure S1.3. Indeed, it shows an interesting pattern that actually helps relate it to our discussion on the relationships between the speciation rate gradient and the species richness gradient.

------

**Reviewer #2:**

This manuscript, by Herrera-Pérez et al., presents an ambitious global-scale analysis, finding a positive relationship between absolute latitude and speciation rate in freshwater fishes, a pattern primarily driven by dynamics in the Northern Hemisphere. This challenges the long-held “tropics as cradle” hypothesis, which states that higher tropical diversity is a consequence of faster speciation rates at lower latitudes. The study is definitely in line with recent work in marine fishes and other major groups and can be a potentially high-impact contribution to the field.
The manuscript has three strengths.
1. The use of a near-complete species-level phylogeny combined with a large database of geographic ranges for over 12,000 freshwater fish species provides a good global coverage.
2. The study employs a variety of methodological tools, applying three distinct speciation rate metrics (ADR, BAMM, ClaDS) and a few statistical models (OLS, SAR, GAM, PGLS).
3. By demonstrating an inverse speciation gradient in a major freshwater vertebrate group, the work extends recent, provocative findings from marine systems to the freshwater realm, adding a crucial piece to the puzzle of what drives the global Latitudinal Diversity Gradient (LDG).

R: We sincerely appreciate the reviewer’s encouraging comments and constructive criticism to improve the framing of our study.

Despite these strengths, the authors can use substantial revision in three key areas to better frame the work.
1. Under-theorized Methodological Choices: The results from its diverse analytical toolkit do not sufficiently discuss the underlying assumptions and deeper implications of the chosen methods. One example of that is the well-documented methodological debate surrounding the BAMM method is not mentioned, and the reasons why different metrics (e.g., ClaDS vs. BAMM) spit out quantitatively different results (such as the location of the latitudinal breakpoint) are not explored. This leaves the analysis feeling more like a black box than a transparent and fully justified scientific investigation.

R: We understand the reviewer’s concern and appreciate the opportunity to clarify our approach. By reducing the number of metrics, excluding several versions and transformations of raw metrics, as well as more clearly justifying our analyses and methodological choices, we believe that our revised study is now more transparent and sound.

In addition, we now explicitly describe our reasoning for using the three different speciation rate metrics based on different models in our methods section and later discuss their effect on the observed patterns and their different results, namely the location of the breakpoint. For example, part of our revised text in the methods section now mentions (lines 181-185 and 193-198):

“*The inference of speciation rates is method dependent, requiring applying several methods to evaluate pattern robustness [12]. We considered three such methods, each with different assumptions, to derive tip-level speciation rate metrics that estimate this rate at the ‘tips’ of the phylogeny and thus describe current speciation rates that can be related to the maintenance of currently observed species richness gradients [8].*”

“*The BAMM method uses a reversible-jump Markov chain model to infer the number and location of diversification-rate shifts across branches and thus accommodates rate heterogeneity better than DR [11]. Although the usefulness of BAMM has been debated [12,40,41], it continues to be widely applied. For example, BAMM-derived speciation rate values are already available for all recognized fish species in the Fish Tree of Life website (*[*https://fishtreeoflife.org/*](https://fishtreeoflife.org/)*), where we obtained the values for our considered species.*”

The use of three different metrics to estimate speciation rates is a good step and ensures robustness. However, the discussion can be beyond simply listing that the results are robust because the metrics are highly correlated and instead engage with the reasons why these methods, which are based on quite different assumptions, might converge or diverge in their estimates (e.g., It is important to state that while the DR statistic is a computationally fast and useful proxy for speciation rate, it is also known to have high variance and can incorrectly identify rate heterogeneity even on trees that are simulated under a constant-rate process, due to the stochastic nature of branching events. Also, it has been commonly misinterpreted in the literature as a measure of net diversification rate, when it is a much better estimator of the speciation rate component.

R: We agree with the reviewer in that we should discuss the reasons for the different results among the chosen speciation metrics given that they have different assumptions. In our revised version, we now explicitly mention that these metrics have different assumptions and are, in fact, “inherently unreliable” (Martínez-Gómez et al., 2023 EvolLett). However, as the reviewer highlights and Martínez-Gómez et al. (2023) conclude, using different metrics is a good step to ensure robustness (which we know acknowledge more strongly in our revised text, lines 181-185).

In our case, using these three metrics based on different methods allowed us to confirm the existence of a latitudinal speciation gradient that was clearly evident at the assemblage-level and absent at the species-level, with these findings being robust to the choice of metric. The main difference among the metrics was the location of the breakpoint in the assemblage-level analysis and this can be related precisely to the methodological differences among them. Following the reviewer’s suggestion, we now explicitly discuss our findings and their differences in light of the methodological properties of each metric. For example, the revised text now includes a new paragraph exclusively dedicated to this issue replicated below (Please, see lines 502-514):

“*Despite revealing a consistent pattern of higher speciation rates towards higher latitudes at the assemblage level, the location of the latitudinal breakpoint varied depending on the speciation rate metric. This can be related to the metrics’ methodological differences. For example, BAMM-derived (e.g., λTC) estimates identify more discrete shifts along the phylogeny compared to both DR and ClaDS [11,12], which can explain its higher latitudinal breakpoint as mainly driven by particular clades with large diversification shifts that belong to the same macroevolutionary regime (e.g., Salmoniformes; [39]). In contrast, DR and ClaDS estimates provide more heterogeneous rates as they accommodate higher rate variation along the phylogeny, especially ClaDS that is capable of identifying many small and frequent shifts in diversification regimes [42]. As a result, under DR and mainly ClaDS, more lineages beyond those belonging to large and discrete macroevolutionary regimes show high speciation rates and push the breakpoint to lower latitudes compared to BAMM given their particular geographic distributions extending to such latitudes (e.g., Cypriniformes).*”

The reviewer is also correct in that all of these tip-level metrics are better estimators of speciation rate than diversification. We explicitly acknowledge this since the introduction, stating it again in our methods and basing all of our description, discussion and interpretation on speciation dynamics instead of diversification.

The manuscript reports a high correlation (Pearson's r>0.8) between ADR and the model-based metrics. This is an interesting finding in itself, as some studies on other taxa, such as birds, have found it to be uncorrelated with BAMM-derived speciation rates. This discrepancy warrants a brief mention in the discussion. It could be speculated that the more pronounced and discrete rate shifts in the fish phylogeny (e.g., the explosive radiations of cichlids and salmonids) create a stronger signal that is consistently picked up by both model-based and non-model-based methods, leading to higher concordance than in clades with more subtle rate variation.

R: Thanks for the suggestion. Indeed, there is considerable discussion around speciation rate estimates derived from different metrics and thus our finding of a high correlation among them is interesting in itself. We particularly appreciate the reviewer’s explicit suggestion to discuss this finding, which we have now used and included in our revised text. Please, see lines 519-537.

“*More importantly, this finding results from clade-specific effects such as the disproportionate influence of the hyper-diverse and regionally concentrated Cichliformes in tropical zones, which comprise several species with the highest speciation rates. Indeed, despite other groups showing high speciation rates outside the tropics, such as Salmoniformes that are restricted to the northern hemisphere and the more widely distributed Cypriniformes and Clupeiformes, the overall species-level pattern is balanced by clades with high speciation rates in both tropical and temperate latitudes ultimately leading to the absence of a latitudinal gradient in speciation rates. This finding was consistent among speciation rate metrics, which at the species-level showed high correlations (Pearson’s r>0.8) perhaps due to the strong signal left by the high-speciating clades that is consistently picked up by all metrics despite their methodological differences and thus leading to their concordance. In contrast, this balancing between tropical and temperate high-speciating clades at the species-level does not erase the latitudinal gradient observed at the assemblage-level. This is mainly because the effect of high-speciating Cichliformes lineages in tropical latitudes is countered by the high species richness and thus co-occurrence of many other lineages from other clades exhibiting lower speciation rates at these same latitudes, whereas the high-speciating lineages at the northern temperate regions co-occur with a lower number of lineages that on average also have high speciation rates (Fig. 3).*”

To provide this missing context and elevate the methodological discussion, the inclusion of a summary table is strongly recommended where the authors list the metric, core principles, key assumptions, and documented strengths and biases.

R: We thank the reviewer for this suggestion. Although, as mentioned in the previous responses, we now include an explicit discussion of our robust findings and their subtle differences to the use of different metrics based on different methods, we decided not to include a table with the characteristics of these different metrics. This was mainly because providing such information would require a more comprehensive analysis under different conditions (e.g., phylogenies reconstructed under different conditions and considering different combinations of known processes, thus simulated/controlled phylogenies), which is clearly beyond the scope of our study. Still, we do describe some of the metrics’ characteristics in our methods sections and then briefly while discussing our findings from these metrics.

2. Problematic Interpretation of Tropical Patterns: The manuscript's narrative frames the exceptionally high speciation rates of certain tropical clades, most notably the Cichliformes, as confounding "noise" that must be statistically removed to reveal a ‘general’ underlying pattern of increasing speciation with latitude. This interpretation represents a significant conceptual weakness. These hyper-diverse clades are not an anomaly obscuring the true signal; they are a primary signal of one of the most powerful modes of diversification on the planet—explosive adaptive radiation. Treating them as outliers to be discarded rather than a distinct macroevolutionary regime to be explained and contrasted with temperate patterns weakens the study's explanatory power. I am not a fish biologist but think this might be an important point to think about.

R: We agree with the reviewer in that this is an important point and we have tried to clarify our approach in the revised version. Our goal was not to neglect the importance of Cichliformes nor consider them as noise, but instead consider their disproportionate effect on the evaluated pattern, which was evident when we excluded them from the whole-class analysis. In fact, we originally considered and discussed both patterns with and without Cichliformes in our previous version. Nevertheless, we agree with the reviewer (and the other reviewers) in that this can be considered conceptually weak, mainly because Cichliformes are not an “anomaly obscuring the true signal” but part of the phenomenon of interest. Therefore, in our revised version we only kept and discussed the analysis/results considering Cichliformes and thus freshwater fishes from the whole class Actinopterygii.

3. Critical Omission of Biodiversity Shortfalls: The manuscript's analysis and conclusions assume that the current taxonomy of freshwater fishes is largely accurate. However, it does not address the well-documented Linnean (undescribed species) and Darwinian (unknown evolutionary relationships) shortfalls, which are particularly acute in the tropics. The high and increasing rate of discovery of cryptic species in tropical freshwater systems, especially in South America and Asia, means that many taxa represented as single, long terminal branches in the phylogeny (implying low speciation rates) may in fact be diverse, rapidly speciating complexes. This systematic underestimation of tropical speciation rates represents a major unaddressed caveat that could plausibly create an artificial inverse gradient, potentially undermining the manuscript's central conclusion.

R: The reviewer raises an important issue: biodiversity knowledge shortfalls. Indeed, different types of knowledge shortfalls that “beset biodiversity knowledge” (Hortal et al. 2015 AREES) can bias our results. In our previous version, we explicitly acknowledged some of these shortfalls (e.g., Wallacean and Darwinian) but failed to discuss them more clearly and robustly. In our revised version, we now do so by considering the aspects suggested by the reviewer and expanding those already mentioned in our previous version by re-writing our last paragraph before our conclusions. The revised paragraph now reads (lines 549-567):

“*Although we found consistent patterns robust to methodological differences of speciation rate metrics, there are other aspects that could bias our results. In particular, the pervasive knowledge shortfalls that hinder our understanding of biodiversity patterns in general (Hortal et al., 2015) and those of fishes in particular (Miqueleiz et al., 2020; Freitas et al., 2020). For example, it is well known that taxonomic diversity is better documented in temperate regions than in tropical regions [71], thus a higher Linnean shortfall in the tropics that is particularly acute for Neotropical fishes given the high number of potential undescribed species (Albert et al., 2020; Birindelli & Sidlauskas 2018[R1] ). Combined with the Darwinian shortfall, regarding our incomplete information on the evolutionary relationships among species (Hortal et al., 2015) and recently recognized for fishes (Soares et a., 2023[R2] ), speciation rates in the tropics may in fact be higher than the ones documented in our study (Tavares et al., 2024). In the same vein, the Wallacean shortfall regarding incomplete knowledge on species' geographic distributions (Hortal et al., 2015) can further obscure patterns mainly at the assemblage level where estimates are derived from the complete ranges of all species across space. Notwithstanding these shortfalls, we found consistent patterns between our two data sets, one considering about 78% (12,557 species) of all described species of freshwater Actinopterygii (the complete dataset) and the molecular dataset with less than half (5,242) of all freshwater fish species. Continued taxonomic revision, expanded geographic sampling, and more refined phylogenetic hypotheses will certainly warrant a reevaluation of our findings. Therefore, our study provides a valuable baseline for future comparative studies as biodiversity knowledge continues to improve.*”

Addressing these three points through careful revision will make this manuscript better and I would happy to reconsider it again.

R: We thank the reviewer for the extremely useful suggestions and guidance to improve our manuscript. By following their suggestions, we strongly believe that our study is now more robust and provides a clearer message to the readers.

------

**Reviewer #3:**

The manuscript “The latitudinal speciation gradient in freshwater fishes: higher speciation at higher latitudes in the northern hemisphere” examines speciation rate across latitudes for the assemblage-level and species-level of freshwater fish. They used a published study on freshwater ranges and phylogenetic information to conduct their statistical analyses. They employed a range of diversification methods and regression models to investigate patterns across latitudes. By using a variety of methods they were able to evaluate a consistent pattern while acknowledging potential biases associated with each method. They were able to build a comprehensive, detailed study and give an overall robust answer. The manuscript is also well-written with objectives clearly defined. While I believe the study is for the most part well-defined, I do have a couple of minor issues that I believe the authors could respond to and not be determinental to the other research.

R: We thank the reviewer for the positive comments and clear suggestions to improve the robustness and clarity of our study.

I was confused on the extent of the study. For the most part it seems to be a global study. While LDG is a pattern observed globally, initially, I thought this study would focus on a more specific region. They do include “across the globe” in the sentence regarding the addition of phylogenetic structure to the assemblage-level and species-level patterns. I would suggest adding that this is a global analysis either in the first or second sentence of the last paragraph of the introduction.

R: Thanks for the suggestion. We now explicitly state that ours is a global study in a couple of instances within the last paragraph of our introduction. For example, lines 125 and 196.

By using species range maps that use convex polygons with Hydrobasin layer level 8, are you overestimating species presence, and how does this affect your results? Would this affect the estimate for the latitude midpoint for each species?

R: The reviewer raises an interesting point. Strictly speaking, all types of range maps built to describe the geographic distribution of species are effectively creating an “extent of occurrence” (i.e., “*the area area which lies within the outermost geographic limits to the occurrence [records] of a species*” Gaston & Fuller, 2009 JApplEcol) and thus necessarily overestimate the species presence, even if only by a little, compared to the observed occurrence records. As such, this procedure can certainly affect the estimation of the species’ latitudinal midpoint, but this potential bias necessarily arises with any method of range map construction and even if only using occurrence records depending on their spatial aggregation. Robustly evaluating such bias would require different methods and assumptions and thus a sound methodological study, which would deviate us from our main message. Still, using range maps to study biodiversity gradients is considered standard practice in macroecology (Vilela & Villalobos, 2015 MEE; Diniz-Filho, 2023 *The Macroecological Perspective*, Springer Press). Indeed, all the relevant studies on fish macroecology and macroevolution in which we based our motivation of our study and discussion of our findings, namely García-Andrade et al. (2023, ProcB) and Rabosky et al. (2018, Nature), used these same type of range maps and the same grid-cell resolution than our study (111 km2 or 1º lat-long), thus allowing fair comparison between our findings and those of the relevant literature.

More specifically, range map polygons allow estimating the geographic distribution of all species of interest, under the same protocol, even when their occurrence records vary in scarcity, incompleteness and/or biases (García-Roselló et al., 2015; Valencia-Rodríguez et al., 2025 FreshwaterBiol, for studies on fishes). In our case, the use of Hydrobasin layer level 8 to create the range maps further allowed us to delimit range boundaries along the actual spatial units within which freshwater fish species occur and move (i.e., the hydrological sub-basins), instead of simply considering imaginary boundaries based on geometry (Valencia-Rodríguez et al., 2025).

In our revised version, we now mention this standard practice in macroecology and the use of the same type of range maps in the relevant studies that relate to ours. For example, when describing this part of our methods section, the added text now reads (lines 152-155):

“*In fact, using such range maps to evaluate biodiversity gradients at large spatial scales is the standard macroecological practice [35], which recently started to be more widely applied to study such gradients for both marine [5] and freshwater fishes [15].*”

Do the 111 x 111 km grid cells overestimate species presence because of the convex polygons in assemblage-level analysis

Why 111 x 111 km resolution grid? If I were to reproduce this study with another organism, I would like to know how that resolution would affect my study.

R: Yes. Using this (or in fact any other) resolution will necessarily overestimate the species presence beyond the actual occurrence records given the use of range maps (‘extents of occurrence’). However, this bias is not idiosyncratic to individual species and thus is replicated across all of them. More importantly, several studies (Hurlbert & Jetz, 2007 PNAS; Hawkins et al., 2008 FoliaGeobot; Keil & Hawkins, 2009 BiodivCons) have shown that the use of range map polygons, such as ours, is best supported by actual occurrence records (ie., show the same spatial patterns) at resolutions of 1º and 2º lat-long at broad spatial scales such as regions, continents, and the globe. Indeed, the 1º (or 111 km2) resolution has been the one used in the most recent literature on fish macroecology (García-Andrade et al., 2023; Rabosky et al. 2018) and the ones we used to base our motivations and discussion of our findings, thus allowing a fair comparison of our study with the relevant literature.

Finally, the reviewer raises a valid claim: “to know how that resolution would affect my study”. Indeed, this was the original question posed by the seminal studies evaluating such an effect and they concluded that the most appropriate resolution(s) when using range map polygons, regardless of the taxa, is around 1º and 2º lat-long (Hurlbert & Jetz, 2007 PNAS).

In our revised version, we now explicitly mention that our choice of grid-cell resolution follows standard practice when using range map polygons and that this choice is congruent with the relevant literature on fish macroecology. For example, the revised text now reads (lines 219-226):

“*This resolution is recommended when using geographic ranges to represent biodiversity gradients at large spatial scales, as they provide similar patterns than those based on occurrences but allowing larger coverage of species and spatial extent [44]. Importantly, recent studies on fish macroecology such as those describing the LDG of freshwater fishes [15] and the latitudinal speciation gradient of marine fishes [5] have used this same resolution and based their analyses on geographic ranges, allowing us to discuss our findings in light of these relevant studies.* ”

The analyses conducted at the species-level produces a very low (but significant) R2, especially compared to the assembly-level analyses. Is this a product of speciation rate or the inclusion of so many (1000+) species.

R: The reviewer is correct. Indeed, the low effect size in the species-level analysis is lower than the one of the assemblage-level analysis. We believe that this finding is of course a product of the speciation rate per species, which is the actual trait being measured, but more importantly a consequence of the variation in such rates across the whole set of species that includes members of different lineages with distinct evolutionary history/dynamics. As such, this is inherently related to the number of included species, comprising members of all major lineages of actinopterygian freshwater fishes that can certainly increase variation. Still, this was precisely our goal, to describe the latitudinal speciation rate gradient when considering all of these species and reveal their spatial structure, which allowed us to compare it against the pattern observed for their marine counterpart as shown by Rabosky et al. (2018).

In our revised version, we now include some of these explanations into our discussion section to express our interpretations more clearly. For example, lines 519-537 now mention:

“*More importantly, this finding results from clade-specific effects such as the disproportionate influence of the hyper-diverse and regionally concentrated Cichliformes in tropical zones, which comprise several species with the highest speciation rates. Indeed, despite other groups showing high speciation rates outside the tropics, such as Salmoniformes that are restricted to the northern hemisphere and the more widely distributed Cypriniformes and Clupeiformes, the overall species-level pattern is balanced by clades with high speciation rates in both tropical and temperate latitudes ultimately leading to the absence of a latitudinal gradient in speciation rates. This finding was consistent among speciation rate metrics, which at the species-level showed high correlations (Pearson’s r>0.8) perhaps due to the strong signal left by the high-speciating clades that is consistently picked up by all metrics despite their methodological differences and thus leading to their concordance. In contrast, this balancing between tropical and temperate high-speciating clades at the species-level does not erase the latitudinal gradient observed at the assemblage-level. This is mainly because the effect of high-speciating Cichliformes lineages in tropical latitudes is countered by the high species richness and thus co-occurrence of many other lineages from other clades exhibiting lower speciation rates at these same latitudes and increasing rate variation, whereas the high-speciating lineages at the northern temperate regions co-occur with a lower number of lineages that on average also have high speciation rates with lower variation (Fig. 3).*”
